# Thoracic Pyogenic Spondylitis Misdiagnosed As Osteoporotic Compression Fracture Status Post Vertebral Augmentation With Resultant Paraplegia: A Case Report

**DOI:** 10.7759/cureus.63497

**Published:** 2024-06-30

**Authors:** Po-Wei Chen, Ping-Chuan Liu, Chin-Cheng Lee, Chee-Tat Lam

**Affiliations:** 1 Department of Neurosurgery, Neurological Institute, Taipei Veterans General Hospital, Taipei, TWN; 2 Department of Pathology, Shin Kong Wu Ho-Su Memorial Hospital, Taipei, TWN; 3 Department of Neurosurgery, Shin Kong Wu Ho-Su Memorial Hospital, Taipei, TWN

**Keywords:** complication, vertebroplasty, vertebral augmentation, misdiagnosis, pyogenic spondylitis

## Abstract

This paper describes a case of serious complications following vertebral augmentation resulting from a misdiagnosis of pyogenic spondylitis as osteoporotic compression fracture (OCF). A 56-year-old female with systemic lupus erythematosus underwent vertebral augmentation following a diagnosis of T10 OCF based on plain film analysis. Note that preoperative computed tomography (CT) and magnetic resonance imaging (MRI) were not performed.

One day after vertebral augmentation, the patient experienced a recurrence of low back pain with fever and paraplegia. MRI findings revealed paravertebral and epidural soft tissue over T9 and T10 with cord compression. Subsequent laminectomy of T9 and T10 revealed devitalized lamina, epidural abscess, and granulation tissue. Pathological analysis indicated a combination of acute and chronic inflammation. A pus culture identified *Staphylococcus aureus*, indicative of pre-existing pyogenic spondylitis. Further revision surgery was performed at another hospital. The patient remained in a paraplegic state one year after surgery.

Infectious spondylitis often manifests with nonspecific symptoms similar to those of compression fracture, and plain radiographs are insufficient to differentiate between the two, often leading to misdiagnosis and mistreatment. Nonetheless, many practitioners base preoperative planning solely on plain film imaging. We advocate the routine usage of CT and/or MRI for patients diagnosed with compression fractures, particularly for immunocompromised individuals.

## Introduction

In an aging society, osteoporotic compression fractures (OCFs) are commonly encountered diseases. Among elderly population and postmenopausal women, the incidence of OCFs is approximately 20% [1]. OCFs could be managed conservatively initially. However, if conservative treatment fails and back pain persists, vertebral augmentation techniques, such as vertebroplasty, balloon kyphoplasty, or vertebral augmentation with implant (instrumented kyphoplasty), should be considered [2,3]. It is estimated that more than 20,000 vertebral augmentations are performed in the United States each year [4]. The incidence of infection following vertebral augmentation is 0.3% [5,6]. Although the risk is low, once developed, it could lead to serious results including neurological deficit and even death [5,6]. We reported a rare case with an initial diagnosis of OCFs receiving vertebral augmentation, followed by rapidly progressive paralysis within one day. In this case, pre-existing pyogenic spondylitis is highly suspected.

## Case presentation

A 55-year-old female with a history of systemic lupus erythematous presented at an orthopedic clinic with symptoms of low back pain following a fall one month prior. The patient was afebrile, and a neurological examination yielded normal results. Laboratory tests revealed a white cell count of 11,000 cells/µL; however, a differential count and C-reactive protein (CRP) concentration were not obtained (Table 1).

**Table 1:** Laboratory test.

	Prevertebral augmentation	One day after vertebral augmentation	Two months after vertebral augmentation	Reference range
White count (cell count/µL)	11,000	15,300	7,050	4,500-11,000
Segmented form (%)	Not obtained	80.7	56	40-70%
C-reactive protein (mg/dL)	Not obtained	28	<0.3	< 0.3
Erythrocyte sedimentation rate (mm/hour)	Not obtained	85	<20	＜20

**Figure 1:**
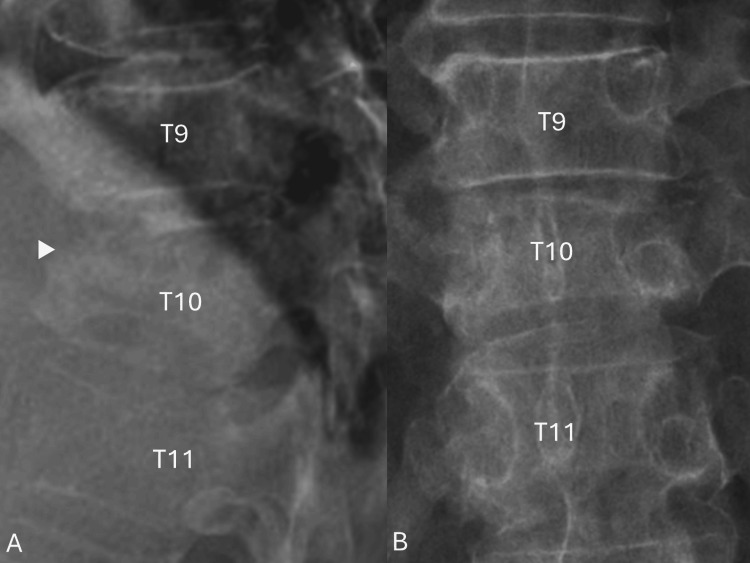
Preoperative plain film of the thoracic spine. (A) Lateral view, anterior wedge fracture, and blurring of the superior end plate of T10 (arrowhead). (B) Anterior-posterior view

Plain film imaging of the thoracic spine revealed a T10 anterior wedge fracture and blurring of the superior end plate (Figures 1A, 1B).

The T-score for dual x-ray absorptiometry was -3.0, indicating osteoporosis. Neither computed tomography (CT) nor magnetic resonance imaging (MRI) was performed. OCFs were suspected as the diagnosis. 

The patient underwent percutaneous vertebral augmentation with an implant placed over T10 using Spinejack (Stryker Corporation, Kalamazoo, MI, USA) with F20 polymethylmethacrylate bone cement (Teknimed, l'Union, France). The prophylactic antibiotic Cefazolin was administered prior to incision. Surgery was completed without immediate complications, and an intraoperative biopsy was not performed.

Postoperatively, the patient reported improved back pain, and the muscle strength in both legs was preserved. However, on the following day, the patient experienced a recurrence of low back pain accompanied by paraplegia and a fever of 40 °C (104°F). Lab tests revealed a white cell count of 15,300/µL with a segment form of 80.7% and CRP of 28 mg/dL (Table 1).

**Figure 2:**
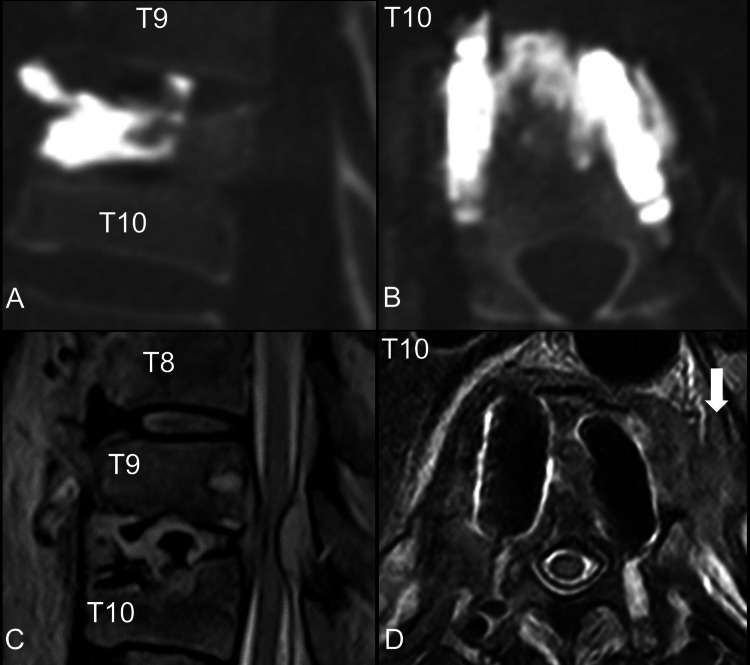
Post-vertebral augmentation images of the thoracic spine. (A) Computed tomography, bone window, sagittal view. (B) Computed tomography, bone window, axial view. (C) Magnetic resonance image, T2 without contrast, sagittal view, T10 level. (D) Magnetic resonance image, T2 without contrast, axial view, T10 level. Abnormal soft tissue was found in the paravertebral space (arrow).

Lumbar spine CT scans without contrast revealed no bone cement leakage; however, abnormal soft tissue was observed in the vicinity of T9 and T10. Lumbar spine MRI without contrast revealed paravertebral and epidural soft tissue over T9 and T10 with cord compression (Figures 2A-2D).

The epidural hematoma was initially suspected; however, infectious spondylitis with epidural abscess could not be ruled out. Empiric Ceftazidime 1g every eight hours and Vancomycin 1g every 12 hours were administered intravenously.

**Figure 3:**
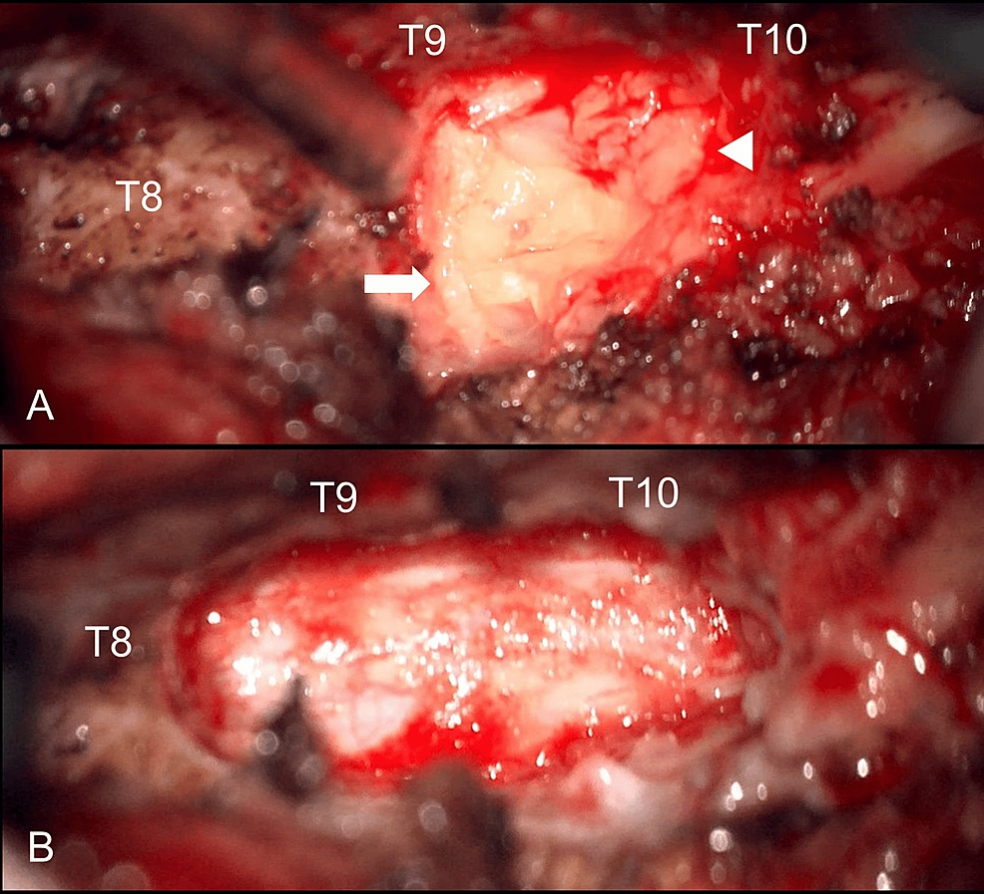
Intraoperative image. (A) Epidural abscess (arrowhead) and granulation tissue (arrow) were identified. (B) The dura was relaxed after decompression.

During decompressive laminectomy over T9 and T10, the neurosurgeon observed soft, devitalized lamina in conjunction with epidural pus-like discharge and tissue granulation (Figures 3A, 3B).

**Figure 4:**
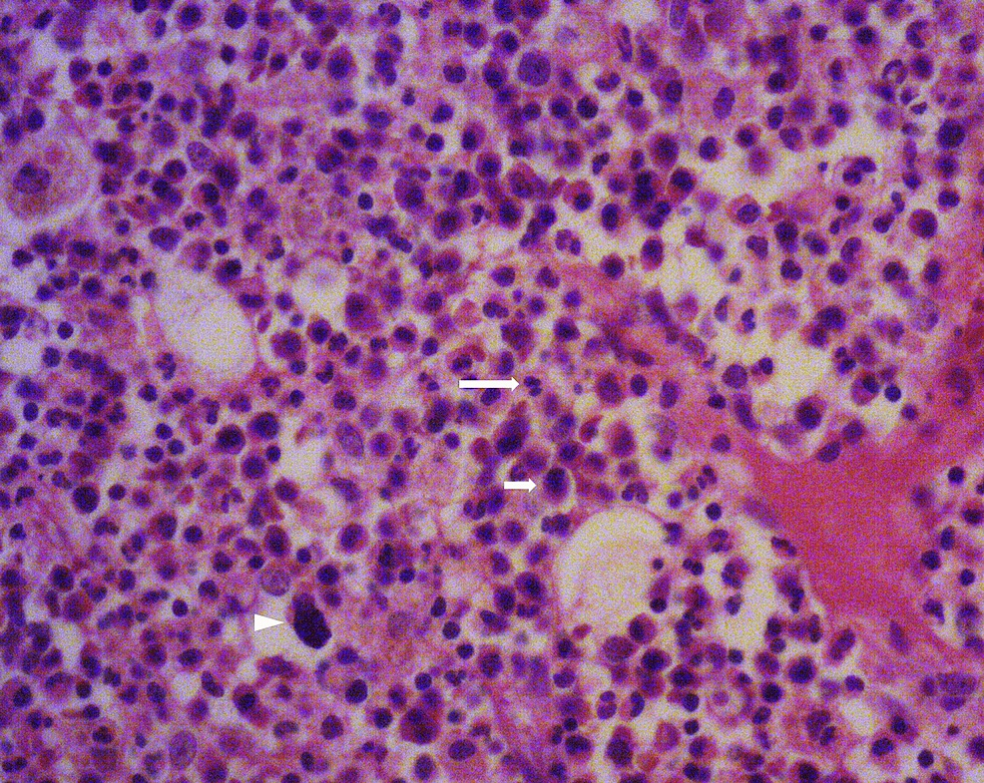
Microscopic images with hematoxylin and eosin staining. Images of the specimen showed necrotic soft tissue and bone tissue with chronic and acute inflammatory cell infiltration. Necrotizing inflammation and osteomyelitis were diagnosed. Original magnification ×400. Macrophage (arrowhead), lymphocyte (short arrow), and neutrophil (long arrow).

Pathological examination confirmed mixed acute and chronic inflammation (Figure 4).

**Figure 5:**
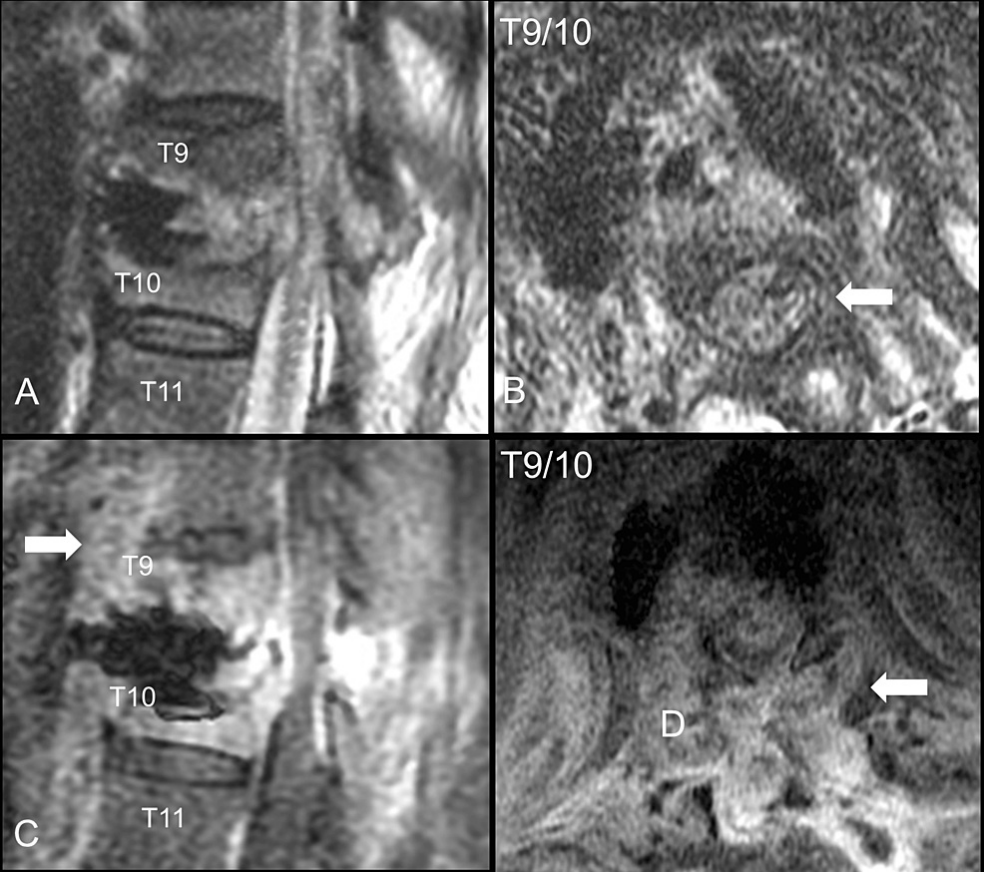
Post-laminectomy magnetic resonance image of the thoracic spine. (A) T2 without contrast, sagittal view. Abnormal soft tissue around the epidural region with spinal stenosis. (B) T2 without contrast, axial view. Dura was not compressed (arrow). (C) T1 with contrast, sagittal view. Paraspinal-enhanced soft tissue was identified (arrow). (D) T1 with contrast, axial view. Paraspinal-enhanced soft tissue was identified (arrow).

Due to extensive infection, instrumentation was not applied. Intraoperative pus culture identified *Staphylococcus aureus*. Vancomycin was maintained and Ceftazidime was discontinued. Follow-up MRI with contrast one week postoperatively revealed enhancement around T9 and T10, as well as stenosis at the T9/10 level (Figures 5A-5D).

Second-stage surgery for implant removal, corpectomy, and fixation was performed at another hospital two weeks later. Upon completion of two months course of Vancomycin, the normalization of inflammation markers was noted (Table 1). Despite intensive rehabilitation, the patient remained in a paraplegic state at a one-year follow-up.

## Discussion

Spondylitis after vertebroplasty is a rare but serious complication. The incidence of pyogenic spondylitis after vertebral augmentation ranges from 0.3% to 1% [6-8]. In a cohort involving 18 patients who underwent revision surgery for spondylitis post-vertebroplasty, the six-month mortality rate was 16.7%, and more than 50% were rendered functionally dependent [6]. A review of the literature revealed that the interval between vertebroplasty and a diagnosis of spondylitis can span several months [6,7,9], which suggests that most cases of spondylitis developed postoperatively.

Few cases of pre-existing infectious spondylitis before vertebral augmentation have been reported. Pola et al. described a 75-year-old male who presented with fever and recurrent back pain two months after T12 vertebroplasty. A re-evaluation of preoperative CT scans revealed atypical endplate destruction, which the authors interpreted as a sign of preoperative infection [10]. Similarly, Yonezawa et al. reported on a 71-year-old male with an L2 vertebral fracture who underwent kyphoplasty. The patient reported recurrent low back pain and fever at two weeks after surgery. The authors suspected pyogenic spondylitis prior to kyphoplasty, based on evidence of atypical isolated lower endplate destruction in preoperative CT scans [11]. However, note that in both of these cases, the evidence for pre-existing spondylitis was not strong, and the chronological sequence of spondylitis and vertebral augmentation could not be confirmed.

To the best of our knowledge, no cases involving the diagnosis of infectious spondylitis within one day of surgery have been reported. In our case, we believe that the probability of pre-existing pyogenic spondylitis was high, given the short interval between vertebral augmentation and symptoms onset, the intraoperative discovery of granulation, and pathological evidence of chronic inflammation.

Patients with pyogenic spondylitis often present with nonspecific symptoms, and misdiagnosing pyogenic spondylitis as OCFs can have disastrous consequences. A thorough evaluation must be conducted to exclude the possibility of infection prior to vertebral augmentation.

Risk factors for infectious spondylitis include long-term steroid use, diabetes mellitus, renal or liver disease, malnutrition, substance abuse, and HIV infection [12]. Note that fever occurs in only 12.5% of infectious spondylitis cases [13]. Moreover, plain film analysis lacks the sensitivity required for the detection of initial indicators (e.g., endplate blurring) or later manifestations (e.g., a decrease in disc height and/or bony destruction) [14]. However, those signs are not uncommon in cases of OCF [15].

Fujiwara et al. reported that endplate injury occurs in 61% of OCFs, and that involvement of the inferior endplate alone is atypical [16]. Yonezawa proposed inferior endplate involvement as a potential indicator of infectious spondylitis [11]. CT scans provide a clear indication of end plate and bone erosion [15], while MRI is the modality of choice for diagnosing pyogenic spondylitis, with a reported sensitivity of 96% [17].

Guidelines published by the American College of Radiology suggest the use of CT or MRI without contrast for all cases of new symptom compression fracture, including those without signs of infection or malignancy [18]. In our case, no signs of infection were initially detected; however, the patient had a history of systemic lupus erythematosus and long-term steroid use, both of which predispose patients to infection. This case was further complicated by the fact that preoperative plain film findings are insufficient to differentiate between infectious spondylitis and OCF. Unfortunately, CT, MRI, or biopsy was not performed to confirm the diagnosis prior to vertebral augmentation.

## Conclusions

Infectious spondylitis often manifests with nonspecific symptoms similar to those of OCF, and plain radiographs are insufficient to differentiate between the two, leading to misdiagnosis and mistreatment. Nonetheless, many practitioners base preoperative planning solely on plain film imaging. We advocate the routine usage of CT and/or MRI for patients diagnosed with compression fractures, particularly for immunocompromised individuals.

## References

[1] Ballane G, Cauley JA, Luckey MM, El-Hajj Fuleihan G (2017). Worldwide prevalence and incidence of osteoporotic vertebral fractures. Osteoporos Int.

[2] De Leacy R, Chandra RV, Barr JD (2020). The evidentiary basis of vertebral augmentation: a 2019 update. J Neurointerv Surg.

[3] Beall D, Lorio MP, Yun BM, Runa MJ, Ong KL, Warner CB (2018). Review of vertebral augmentation: an updated meta-analysis of the effectiveness. Int J Spine Surg.

[4] Laratta JL, Shillingford JN, Lombardi JM (2017). Utilization of vertebroplasty and kyphoplasty procedures throughout the United States over a recent decade: an analysis of the Nationwide Inpatient Sample. J Spine Surg.

[5] Park JW, Park SM, Lee HJ, Lee CK, Chang BS, Kim H (2018). Infection following percutaneous vertebral augmentation with polymethylmethacrylate. Arch Osteoporos.

[6] Liao JC, Lai PL, Chen LH, Niu CC (2018). Surgical outcomes of infectious spondylitis after vertebroplasty, and comparisons between pyogenic and tuberculosis. BMC Infect Dis.

[7] Zhang S, Wang S, Wang Q, Yang J, Xu S (2021). Debridement and corpectomy via single posterior approach to treat pyogenic spondylitis after vertebral augmentation. BMC Musculoskelet Disord.

[8] Robinson Y, Tschöke SK, Stahel PF, Kayser R, Heyde CE (2008). Complications and safety aspects of kyphoplasty for osteoporotic vertebral fractures: a prospective follow-up study in 102 consecutive patients. Patient Saf Surg.

[9] Schofer MD, Lakemeier S, Peterlein CD, Heyse TJ, Quante M (2011). Primary pyogenic spondylitis following kyphoplasty: a case report. J Med Case Rep.

[10] Pola E, Autore G, Pambianco V, Formica VM, Colangelo D, Nasto LA (2016). A particular case of pyogenic spondylodiscitis misdiagnosed as a vertebral fragility fracture and erroneously treated with balloon kyphoplasty. Spine J.

[11] Yonezawa N, Tokuumi Y, Komine N (2021). Simultaneous-onset infectious spondylitis with vertebral fracture mimicking an acute osteoporotic vertebral fracture erroneously treated with balloon kyphoplasty: illustrative case. J Neurosurg Case Lessons.

[12] Cheung WY, Luk KD (2012). Pyogenic spondylitis. Int Orthop.

[13] Butler JS, Shelly MJ, Timlin M, Powderly WG, O'Byrne JM (2006). Nontuberculous pyogenic spinal infection in adults: a 12-year experience from a tertiary referral center. Spine (Phila Pa 1976).

[14] Varma R, Lander P, Assaf A (2001). Imaging of pyogenic infectious spondylodiskitis. Radiol Clin North Am.

[15] Dhodapkar MM, Patel T, Rubio DR (2023). Imaging in spinal infections: current status and future directions. N Am Spine Soc J.

[16] Fujiwara T, Akeda K, Yamada J, Kondo T, Sudo A (2019). Endplate and intervertebral disc injuries in acute and single level osteoporotic vertebral fractures: is there any association with the process of bone healing?. BMC Musculoskelet Disord.

[17] Modic MT, Feiglin DH, Piraino DW, Boumphrey F, Weinstein MA, Duchesneau PM, Rehm S (1985). Vertebral osteomyelitis: assessment using MR. Radiology.

[18] Khan MA, Jennings JW, Baker JC (2023). ACR Appropriateness Criteria® management of vertebral compression fractures: 2022 update. J Am Coll Radiol.

